# RNA-based short tandem repeat analysis on the D18S51 locus

**DOI:** 10.17912/micropub.biology.001190

**Published:** 2024-04-24

**Authors:** Seiji Kubo, Keito Amai, Jin Tanaka, Hideki Niimi

**Affiliations:** 1 Department of Clinical Laboratory and Molecular Pathology, Faculty of Medicine, Academic Assembly, University of Toyama, Toyama, Toyama, Japan; 2 Forensic Science Laboratory, Ishikawa Prefectural Police, Kanazawa, Ishikawa, Japan

## Abstract

DNA typing based on short tandem repeat (STR) analysis is an effective forensic method for human identification. Some STRs are contained within the introns of protein-coding genes and are transcribed as pre-mRNAs. However, the possibility of using RNA for STR analysis is yet to be fully explored. Considering that RNA in forensic samples is relatively stable, especially under dry- and low-temperature conditions, we hypothesized that STR information could be obtained from RNA. Here, we investigated the possibility of conducting RNA-based STR analysis using the D18S51 locus as a model.

**Figure 1. RNA-based STR analysis on the D18S51 locus:**
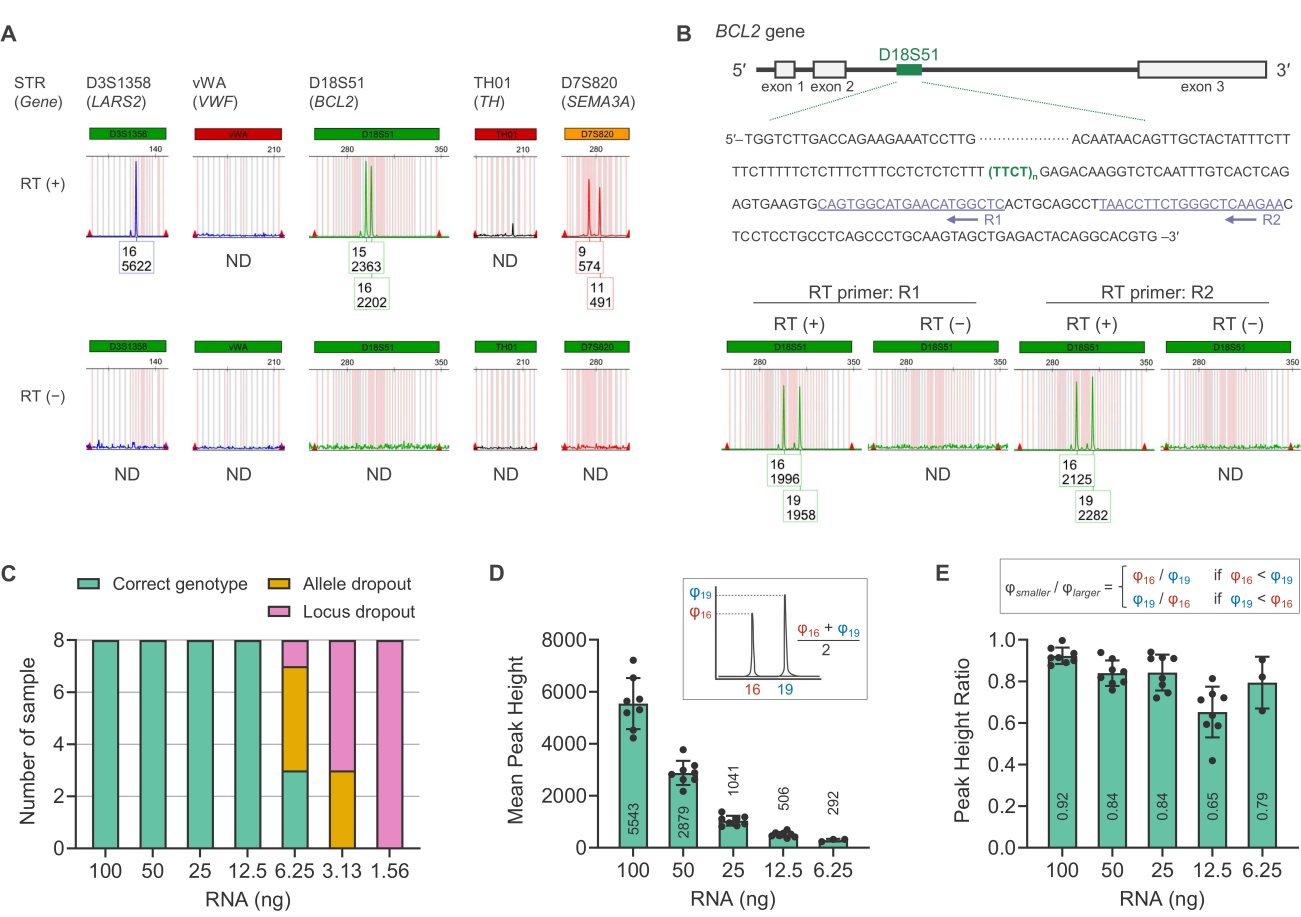
(A) STR analysis on K562 RNA with/without reverse transcription (RT (+/−)). Representative electropherograms are shown. Amplified RNA (cDNA) is detected as fluorescent signals and represented as peaks on the electropherogram. The numbers in the box indicate the repetitions of the repeat unit (upper) and peak height (lower). (B) STR analysis on leukocyte RNA with/without reverse transcription (RT (+/−)). Schematic representation (upper panel) and representative electropherograms (lower panel) are shown. (C–E) Sensitivity test. (C) The number of samples showing the correct genotype, allele dropout, and locus dropout. n = 8 technical replicates. (D) The mean peak height of heterozygote peaks. (E) The ratio of heterozygote peak height. (D, E) Data are expressed as mean ± SD (n = 8 for 100–12.5 ng; n = 3 for 6.25 ng; corresponding to the number of samples that show the correct genotype in (C)).

## Description

In the forensic field, DNA typing based on short tandem repeat (STR) analysis is effective for the human identification of perpetrators and victims. Several commercial STR kits have been validated (Oostdik et al., 2014; Ludeman et al., 2018) and are used in criminal investigations worldwide. For example, the GlobalFiler™ PCR Amplification Kit (Thermo Fisher Scientific) detects 21 autosomal STR and 3 sex markers (Y-chromosome STR, amelogenin locus on X/Y-chromosomes, and insertion/deletion polymorphisms on Y-chromosome).

In addition to DNA profiling, other valuable information can be obtained from forensic samples. Notably, many studies have reported the use of RNA for body fluid identification (Juusola and Ballantyne, 2005; Lindenbergh et al., 2012; Hanson et al., 2018) . Identifying body fluids in forensic samples is crucial for reconstructing the crime scene. It aids in providing insights into how the forensic samples are left. Importantly, RNA is stable in forensic samples, particularly when placed under dry- and low-temperature conditions.

Some STR loci are located within the introns of protein-coding genes. For example, vWA is found in the 40th intron of the von Willebrand Factor ( *VWF* ) gene (Kimpton et al., 1992) ; D18S51 is found in the intron between exons 2 and 3 of the B-cell CLL/lymphoma 2 ( *BCL2* ) gene (Klintschar et al., 2006) ; and TH01 is found in the first intron of the tyrosine hydroxylase ( *TH* ) gene (Polymeropoulos et al., 1991) . This indicates that these STRs are transcribed in the form of pre-mRNAs. Considering the relative stability of RNA in forensic samples, we hypothesized that RNA could be used for STR analysis. In this study, we present the possibility of performing RNA-based STR analysis, using the D18S51 locus as a model.

Initially, we examined whether RNAs were expressed from the STR loci that are generally used in forensic analyses. Using the K562 RNA sample, we synthesized cDNA by random reverse transcription (random hexamer and oligo dT primers), then amplified the cDNA using the commercial STR amplification kit, GlobalFiler™ PCR Amplification Kit (GF kit). STR typing results were detectable at several loci, indicating the presence of STR transcripts ( Fig. 1A ). Of these, we arbitrarily selected the D18S51 locus for subsequent analyses. At the D18S51 locus, two allele peaks, heterozygote peak pattern of “15 and 16” (genotype; the number indicates the repetitions of the repeat unit), were detected. This RNA typing result is consistent with the DNA typing results of a previous study (Szibor et al., 2003) . Because no amplifications were detected in the RT (−) sample, we confirmed that the observed alleles were derived from RNA rather than from residual DNA.

To exclude the possibility that D18S51 RNA ( *BCL2* pre-mRNA) is expressed only in tumors, we tested the normal leukocyte RNA sample. We performed gene-specific reverse transcription using primers R1 or R2 ( Fig. 1B ), followed by PCR using the GF kit. The resulting electropherogram displayed a heterozygote peak pattern of “16 and 19.” Therefore, we verified the feasibility of RNA typing for normal body fluid/tissue samples, such as blood samples. Furthermore, because the cDNA templates were synthesized using gene-specific primers, we concluded that the observed typing results accurately reflected *BCL2* pre-mRNA sequences.

Subsequently, we examined sensitivity using diluted leukocyte RNA samples. The correct genotype (heterozygote peaks) was detectable using 100–12.5 ng of RNA ( Fig. 1C ). Allele dropout, defined as the absence of one of two alleles at a heterozygous locus (Taberlet et al., 1996, 1999) , was observed in 6.25 ng and 3.13 ng of RNA; and locus dropout (the absence of both alleles) was observed in 6.25–1.56 ng of RNA. Similar to conventional DNA-based STR analysis, the decrease in RNA to be reverse transcribed resulted in a decreased peak height ( Fig. 1D ) and an imbalance in the heterozygote peak ( Fig. 1E ). Altogether, the reliable sensitivity was 25 ng of leukocyte RNA. Because 50 ng of RNA is required for body fluid identification using RNA-seq (Hanson et al., 2018) , RNA-based STR analysis has higher sensitivity than RNA-seq analysis. However, we note that the synthesis of cDNA, which is the direct template for STR analysis, is highly dependent on the reverse transcription step. Various factors involving reverse transcription could affect the sensitivity.

In this study, we demonstrated the applicability of pre-mRNAs for STR analysis. RNA-based STR analysis may help interpret DNA typing results that are complicated by DNA mixtures (i.e., DNA from more than one contributor) (Jobling and Gill, 2004; Butler, 2015) . For example, when two biological samples are mixed at different times, STR analysis using relatively stable DNA may show a mixed profile of two individuals; in contrast, STR analysis using RNA that is more susceptible to degradation than DNA may show a single profile from one individual. However, further studies are required to verify whether RNA-based STR analysis can be practically applied. Future studies should investigate (1) general characteristics such as stutter (by-products of STR amplification) (Schlötterer and Tautz, 1992); (2) applicability to forensically-relevant body fluids, mixed samples, and casework samples; and (3) intra- and inter-individual variations in expression levels.

## Methods

*Sample*

Total RNA samples derived from the K562 tumor cell line and normal blood leukocytes were purchased from BioChain (Newark, CA). K562 RNA (50 ng input) was used for random reverse transcription, followed by STR analysis. Leukocyte RNA (50 ng input) was used for gene-specific reverse transcription, followed by STR analysis. For the sensitivity test, leukocyte RNA was 2-fold serially diluted with TE buffer (Thermo Fisher Scientific, Waltham, MA): expected RNA input = 100, 50, 25, 12.5, 6.25, 3.13, and 1.56 ng.

*Reverse transcription*

Reverse transcription with random hexamer and oligo dT primers was performed using the PrimeScript™ RT Master Mix (TaKaRa, Shiga, Japan) on a ProFlex™ PCR System (Thermo Fisher Scientific) according to the manufacturer’s protocol. The synthesized cDNA was 4-fold diluted with TE buffer.

Reverse transcription with gene-specific primers (R1 or R2) was performed using the PrimeScript™ Reverse Transcriptase (TaKaRa) on a ProFlex™ PCR System according to the manufacturer’s protocol. The synthesized cDNA was 4-fold diluted with TE buffer.

*STR analysis*

STR analysis on 10 µL of each cDNA was performed using the GlobalFiler™ PCR Amplification Kit (Thermo Fisher Scientific) on a ProFlex™ PCR System according to the manufacturer’s protocol (29-cycle protocol). The PCR products (1 µL) were mixed with Hi-Di™ Formamide (9.6 µL; Thermo Fisher Scientific) and GeneScan™ 600 LIZ™ dye Size Standard v2.0 (0.4 µL; Thermo Fisher Scientific). Electrophoresis was performed using the 3500xL genetic analyzer (Thermo Fisher Scientific). Data were analyzed using the GeneMapper™ ID-X software v1.4 (Thermo Fisher Scientific) with the peak detection threshold of 175. The mean peak height was calculated by averaging the heterozygote peaks. The peak height ratio was calculated by dividing the lower peak by the higher one.

## Reagents

*Reagents*

**Table:** 

**Reagent**	**Source**	**Identifier**
Total RNA—Human Tumor Cell Line: K562	BioChain	R1255820-50
Total RNA—Human Adult Normal Tissue: Peripheral Blood Leukocyte	BioChain	R1234148-10
PrimeScript™ RT Master Mix	TaKaRa	RR036A
PrimeScript™ Reverse Transcriptase	TaKaRa	2680A
Advantage® UltraPure PCR Deoxynucleotide Mix	TaKaRa	639125
Primers	Eurofins Genomics K.K.	−
TE Buffer	Thermo Fisher Scientific	12090015
GlobalFiler™ PCR Amplification Kit	Thermo Fisher Scientific	4476135
GeneScan™ 600 LIZ™ dye Size Standard v2.0	Thermo Fisher Scientific	4408399
Hi-Di™ Formamide	Thermo Fisher Scientific	4311320

*Primers*

**Table:** 

**Name**	**Sequence (5' to 3')**	**Reference**
R1	GAGCCATGTTCATGCCACTG	STRBase ^a^ (National Institute of Standards and Technology)
R2	TTCTTGAGCCCAGAAGGTTA	STRBase ^a^ (National Institute of Standards and Technology)

^a^ <https://strbase-archive.nist.gov/str_D18S51.htm>
